# Supplementary material for: Anticancer and Structure Activity Relationship of Non-Symmetrical Choline Kinase Inhibitors
Source: Pharmaceutics. 2021 Aug 29;13(9):1360. doi: 10.3390/pharmaceutics13091360 (PMC8464809; doi:10.3390/pharmaceutics13091360)
Supplement: Supplementary file 1 [file pharmaceutics-13-01360-s001.zip › pharmaceutics-1341626-SM.pdf]

pharmaceutics

MDPI

Supplementary Materials: Anticancer and Structure Activity Relationship of Non-Symmetrical Choline Kinase Inhibitors

Santiago Schiaffino-Ortega, Elena Mariotto, Pilar María Luque-Navarro, María Kimatrai-Salvador, Pablo Rios-Marco, Ramon Hurtado-Guerrero, Carmen Marco, María Paz Carrasco-Jimenez *, Giampietro Viola * and Luisa Carlota López-Cara *

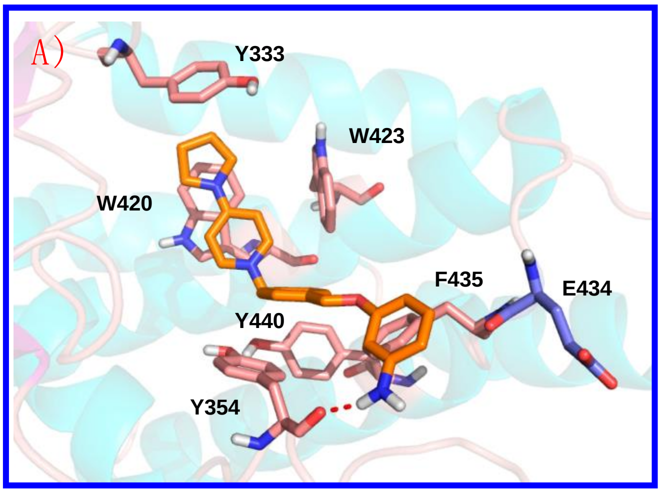

Compound 3a

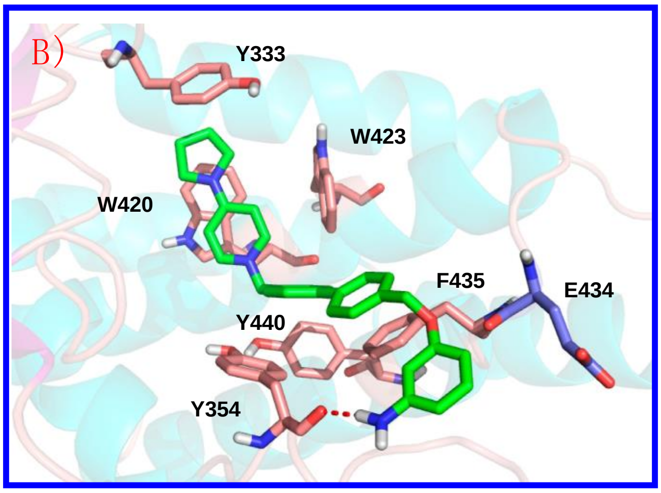

Compound 3b

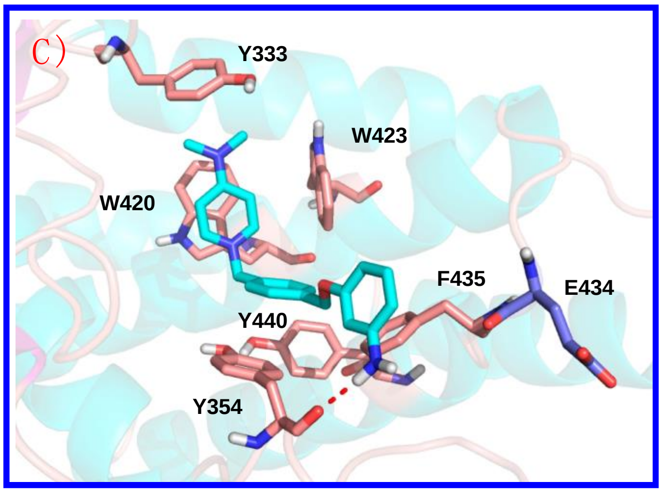

Compound 3e

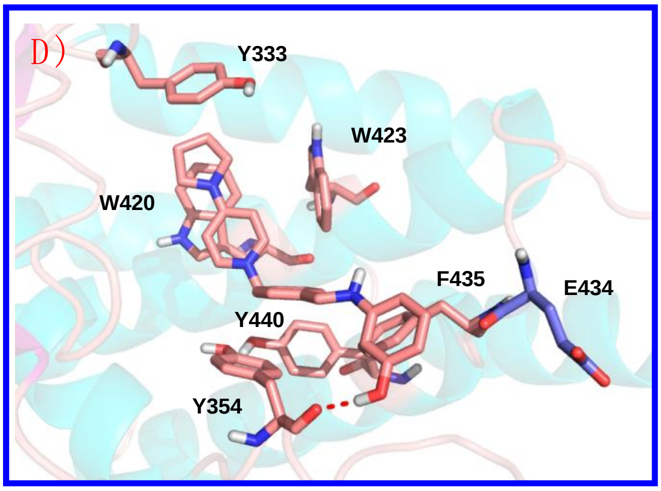

Compound 4a

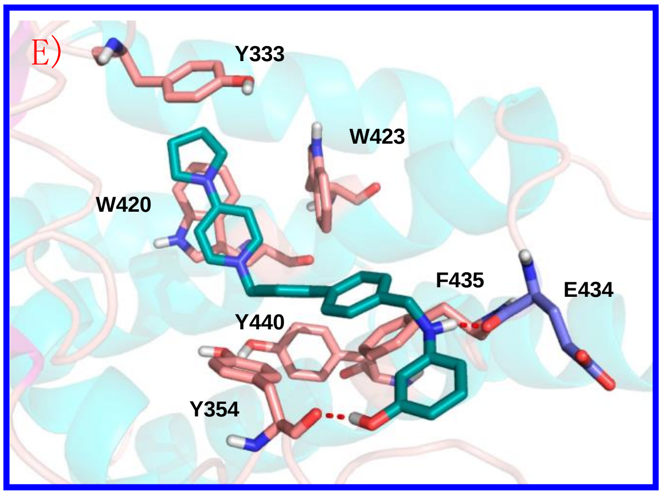

Compound 4b

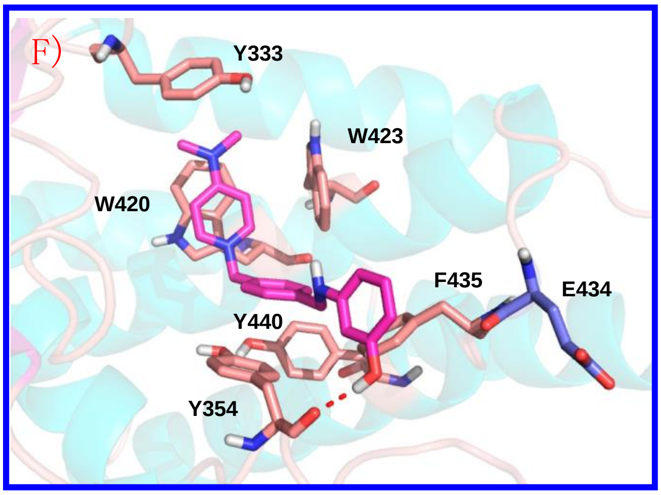

Compound 4e

Figure S1. Poses obtained in the docking studies of compounds **3a** (**A**, carbon atoms in orange colour), **3b** (**B**, carbon atoms in green colour), **3e** (**C**, carbon atoms in cyan colours), **4a** (**D**, carbon atoms in salmon colours), **4b** (**E**, carbon atoms in slate colour) and **4e** (**F**, carbon atoms in magenta colour), inserted into the Cho binding site similarly to of ChoKα1/**6** complex (PDB ID: 4BR3).

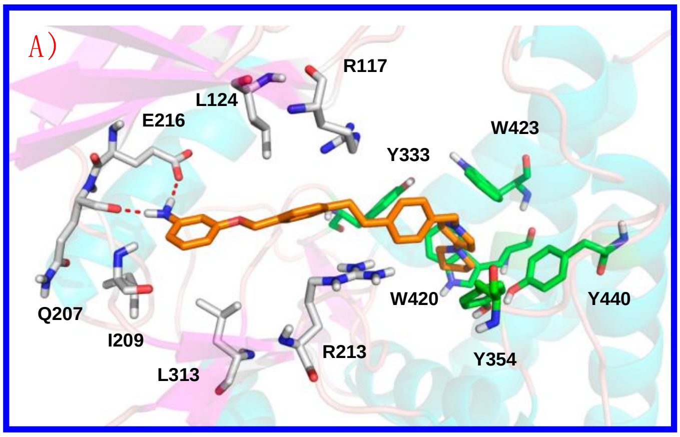

Compound 3c

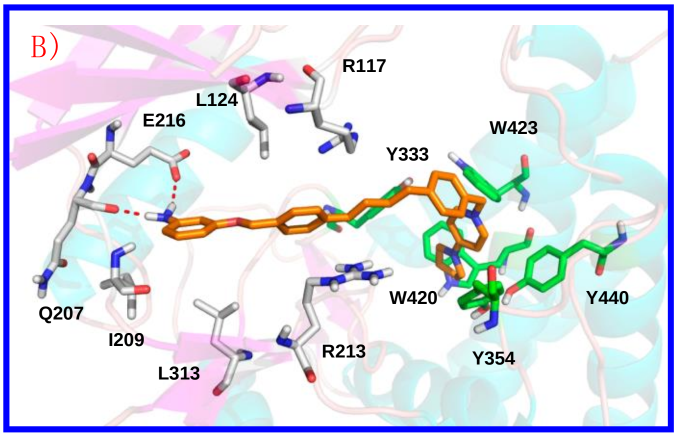

Compound 3d

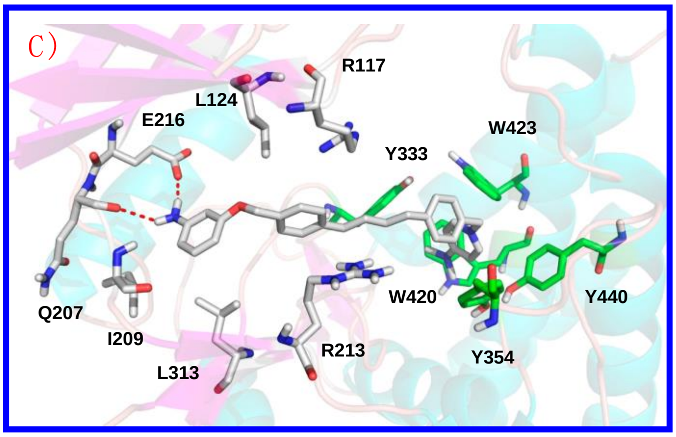

Compound 3h

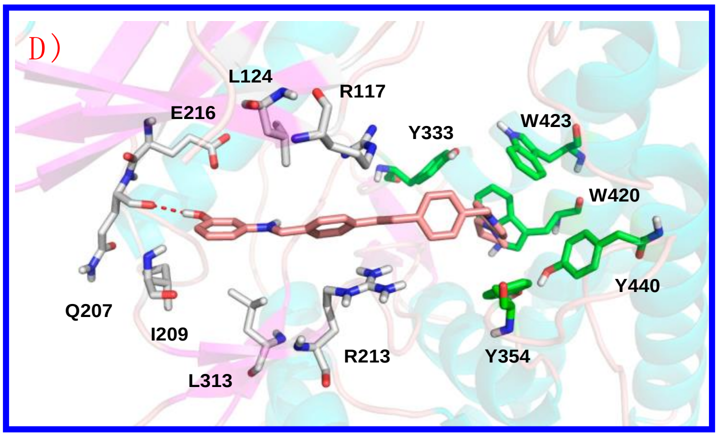

Compound 4c

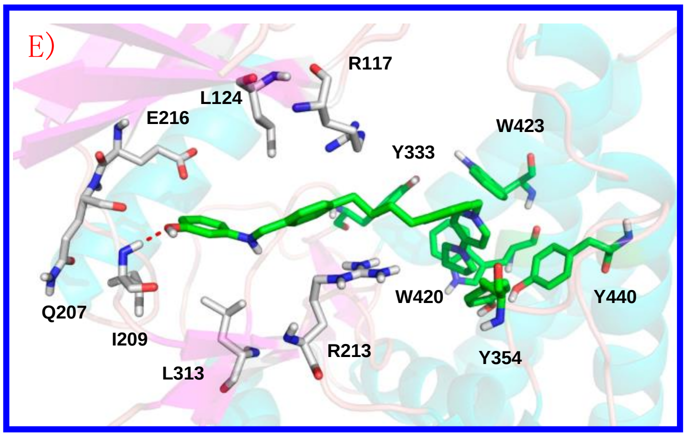

Compound 4d

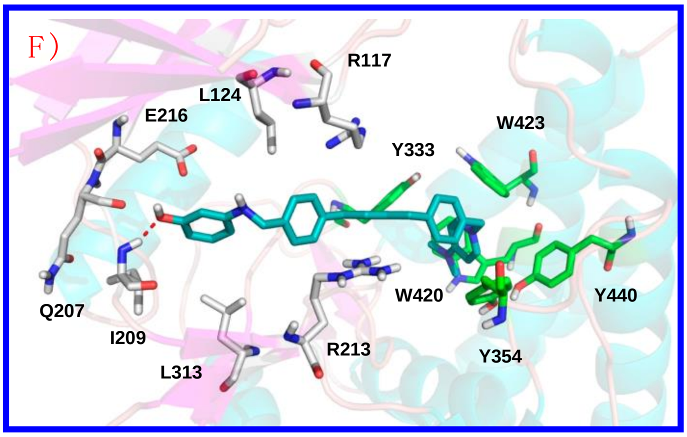

Compound 4h

Figure S2. Poses obtained in the docking studies of compounds **3c** (**A**, carbon atoms in orange colour), **3d** (**B**, carbon atoms in orange colour), **3h** (**C**, carbon atoms in white colours), **4c** (**D**, carbon atoms in salmon colours), **4d** (**E**, carbon atoms in green colour) and **4h** (**F**, carbon atoms in cyan colour), inserted into the ATP and Cho binding sites similarly to ChoK-α1/**5** complex (PDB ID: 3ZM9).
